# CT Features of Saba Senegalensis (a Tropical Fruit) in Patients with Abdominal Pain

**DOI:** 10.5334/jbr-btr.1434

**Published:** 2018-01-04

**Authors:** Ousséini Diallo, Nayi Zongo, Patrice Jissendi Tchofo

**Affiliations:** 1 Yalgado Ouedraogo hospital, Ouagadougou, Burkina Faso, BF; 2 CHU Saint-Pierre, Brussels, BE

**Keywords:** Abdominal pain, Bowel obstruction, Saba senegalensis, Cowries, Phytobezoar

## Abstract

Saba senegalensis (SS) is a well-known and commonly eaten fruit in Western Africa, especially in the rainy season when it is abundant. The ingestion of its seeds may cause abdominal pain and bowel obstruction. This cause might not be recognized by radiologists who are not aware of SS CT features. We thus present the characteristic CT features of SS as found in patients presenting with abdominal pain and incidentally in others. We also discuss the differential diagnosis with the cowries (ornament) and other similar fruits as imaged on CT.

From 2009 through 2015 a series of 14 patients, aged 4–65 years (10 males) were referred to our hospital. The reasons for abdominal/lumbar CT were: abdominal pain only, signs of bowel occlusion, neoplasm, trauma, scoliosis and spondylodiscitis. In all these patients, we found in the bowel, objects appearing as oval-shaped foreign bodies, spontaneously hyperdense with a midline hypodense line along its long axis (Figure 1A, B). The multiplicity, same shape and density of the objects led us to search for something swallowed. In most of the cases we reached the final diagnosis of tropical fruits ingestion after questioning the relatives. Our own investigations including CT scans (using a spiral 16 detectors CT, with 5 mm slice thickness) of the *saba senegalensis* (SS) fruit (Figure 1C, D) drew us to the conclusion that the seeds of SS were the objects seen. They were obviously the cause for abdominal pain and/or bowel obstruction in six cases, while asymptomatic and incidentally found in the others.

**Figure 1:**
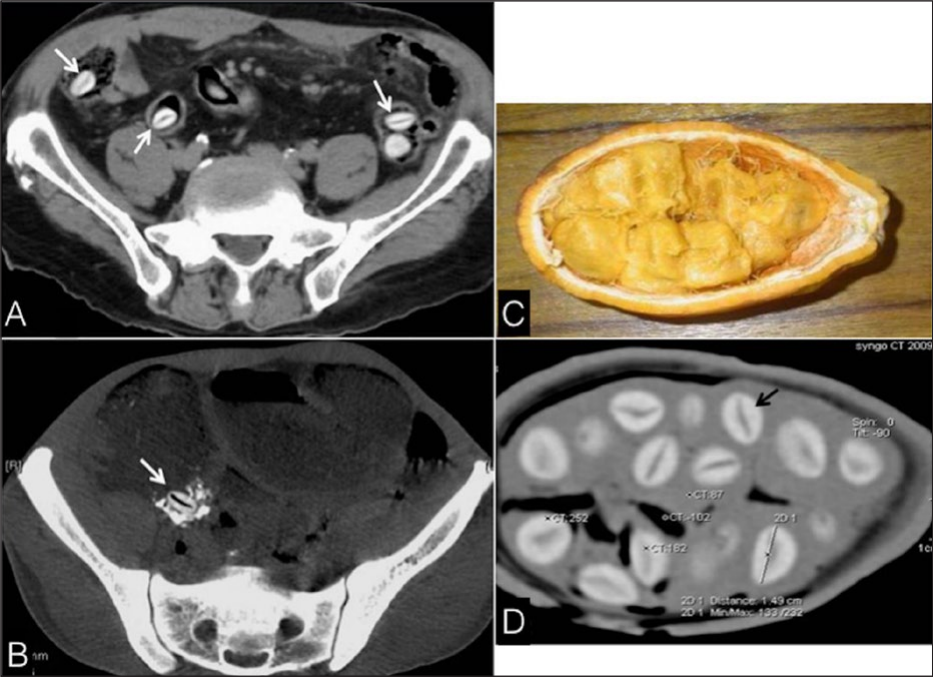
Axial CT images showing **(A)** SS seeds, incidentally found in the bowel (arrows), and **(B)** A seed impacted in the ileocecal valve (arrow), responsible for occlusion; Photograph **(C)** and CT **(D)** of SS; seed (dark arrow): maximum long diameter: 16 mm, high density of the shell (256 HU) and the midline linear hypodensity (92 HU).

## Comment

**Figure 2:**
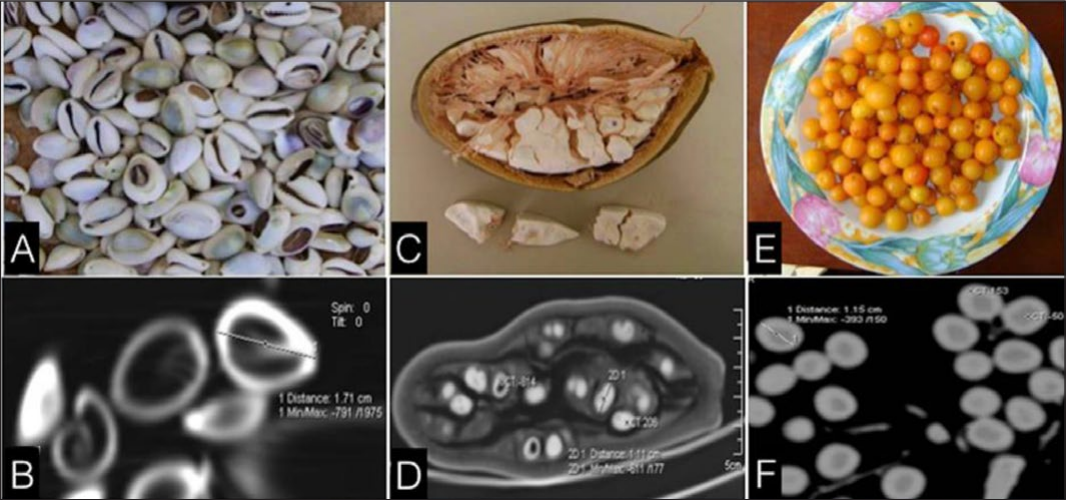
CT differential diagnoses of SS seeds. Photograph/CT of *Cowries* **(A/B)** Maximum diameter of 17 mm, periphery: 1343 HU, core: –1024 HU; *Adansonia digitata* **(C/D)** Maximum diameter of 11 mm, periphery: 206 HU, core: –814 HU; and *Ximenia americana* **(E/F)** Maximum diameter of 12 mm, the periphery: 153 HU, core: –50 HU.

*Saba senegalensis* (SS), also called *Liana goyin*, is a well-known and commonly eaten fruit in Western Africa. The ingestion of its seeds may remain asymptomatic, but can also cause abdominal pain and bowel obstruction. This cause might not be recognized by radiologists who are not aware of SS CT features. Thus, on abdominal CT imaging, the seeds can be found in the gastrointestinal tract, incidentally or in cases of abdominal pain or bowel obstruction. The typical appearance of the seed is a hyperdense shell with a midline dark line. The high density is likely due to the high concentration of calcium and carbohydrates compounds. Gastrointestinal foreign bodies can be identified only if their shape is recognized. SS should not be mistaken for *cowries*, shells originating from the Maldives and used in ancient African societies as a currency for trade but nowadays as an ornament (Figure 2A, B), and other fruits seeds, such as *Adansonia digitata* (Figure 2C, D) and *Ximenia americana* (Figure 2E, F). SS can behave like an obstructive phytobezoar, or reveal an underlying malignant pathology. Small bowel obstruction due to phytobezoars are most common in patients with impaired gastrointestinal motility. The risk of obstruction is significant for a bezoar of 32 mm major diameter [1], which is the double size of SS seeds.

Independent of its size, the location and accumulation of phytobezoars, as well as their chemical composition (may induce an inflammatory response), are responsible for bowel obstruction. Abdominal CT is the imaging modality of choice allowing a good topographical localization of the phytobezoar, but the diagnosis is often made difficult because of their rarity.
